# Pfeiffer syndrome in an adult with previous surgical correction: A case report of CT findings

**DOI:** 10.1016/j.radcr.2021.06.003

**Published:** 2021-07-02

**Authors:** Neil Duggal, Adil Omer, Sandhya Jupalli, Leszek Pisinski, Alan V. Krauthamer

**Affiliations:** Department of Radiology, NYC Health Hospitals, Harlem, NY, USA

**Keywords:** Craniosynostosis, Craniofacial-skeletal-dermatologic dysplasia, Noack, FGF, Pfeiffer, Acrocephalosyndactyly, Le fort, Apert, Cloverleaf

## Abstract

Pfeiffer syndrome, affecting roughly 1 in 100,000 individuals is characterized by acrocephalosyndactyly – the **premature** closure of skull sutures (craniosynostosis). These acrocephalosyndactyly syndromes which are often sporadic de novo but also autosomal dominant in inheritance can be characterized by the fact that they often involve FGFR and TWIST genes. In the presented case, a 27-year old male level three trauma admission displayed skull abnormalities on physical examination that history taking confirmed was the result of pediatric surgically corrected Pfeiffer syndrome. Noncontrast brain CT as part of his trauma work-up revealed characteristic Pfeiffer syndrome imaging pattern of midface hypoplasia, nonvisualization of coronal and sagittal sutures, and a degree of obstructive hydrocephalus. Pfeiffer syndrome requires extensive pediatric surgery often with poor adult follow up. The case presented provides good visualization of characteristic skull abnormalities in a surgically corrected adult. By virtue of imaging an adult, this provides readers with a unique look at the long-term viability and the body's resulting physiological adaptations of the extensive mandatory pediatric surgery these patients undergo.

## Introduction

Pfeiffer syndrome (OMIM 101600, also known as type 5 acrocephalosyndactyly, craniofacial-skeletal-dermatologic dysplasia, or Noack syndrome) is part of a family of syndromes characterized by acrocephalosyndactyly – the premature closure of skull sutures (craniosynostosis) that are separated into five types [1]. First described in 1964 by German geneticist, Rudolf Arthur Pfeiffer who noted eight individuals in three generations of a family with notable abnormalities of head, hands, and feet in an autosomal dominant inheritance pattern. Since his discovery, at least six additional Pfeiffer pedigrees and at least a dozen sporadic cases have been recorded, making Pfeiffer syndrome's incidence rate of 1 in 100,000 individuals [2], [3], [4]. Given the paucity of cases, there is scarce follow up data on patients [1]. Pfeiffer syndrome is genetically heterogeneous and caused by mutations in FGFR1 and FGFR2. It has been noted that both Pfeiffer and Crouzon syndrome, another member of the acrocephalosyndactyly family, are caused by similar pathological variant FGFR2 mutations [5], [6], [7]. Interestingly, there have been no reports of scoliosis in Pfeiffer syndrome, but there have been case reports of scoliosis in Crouzon syndrome, begging the question if the patient's craniosynostosis syndrome has been misclassified [8]. Due to this genetic and phenotypic overlap between Crouzon and Pfeiffer syndrome, patients with the same pathologic variant have been diagnosed as the other in the past. However, there are certain pathologic variants exclusively reported as Pfeiffer syndrome in medical literature [9]. Pfeiffer syndrome is subdivided into three broad phenotypes with overlap. Point mutations in Ser351Cys of FGFR2 meaning cysteine replacement of serine at amino acid codon 351 is associated with a more severe phenotype and decreased survival [10]. Type 1 (classic) is more commonly hereditary with sporadic cases reported while in type 2 and 3 only sporadic cases have been reported except for an exclusive type 2 pedigree. Type 1 is associated with FGFR1 or 2 mutations while type 2 and 3 are exclusively FGFR2 mutations.^2^

## Case report

A 27-year old male presented to the emergency room status post fall from a bike as a trauma admission. Physical examination revealed skull abnormalities consistent with craniosynostosis deformities that had been surgically corrected including underdeveloped midface, widely spaced eyes, and full forehead. Upon history taking it was revealed that the patient had been previously diagnosed with Pfeiffer syndrome. Upon further questioning the patient had undergone extensive craniofacial pediatric surgery and had a history of scoliosis. An emergent CT of the head without intravenous contrast was obtained revealing no evidence of acute intracranial injury abnormality but presence of multiple bony abnormalities with physiologic consequences.

**Fig. 1:**
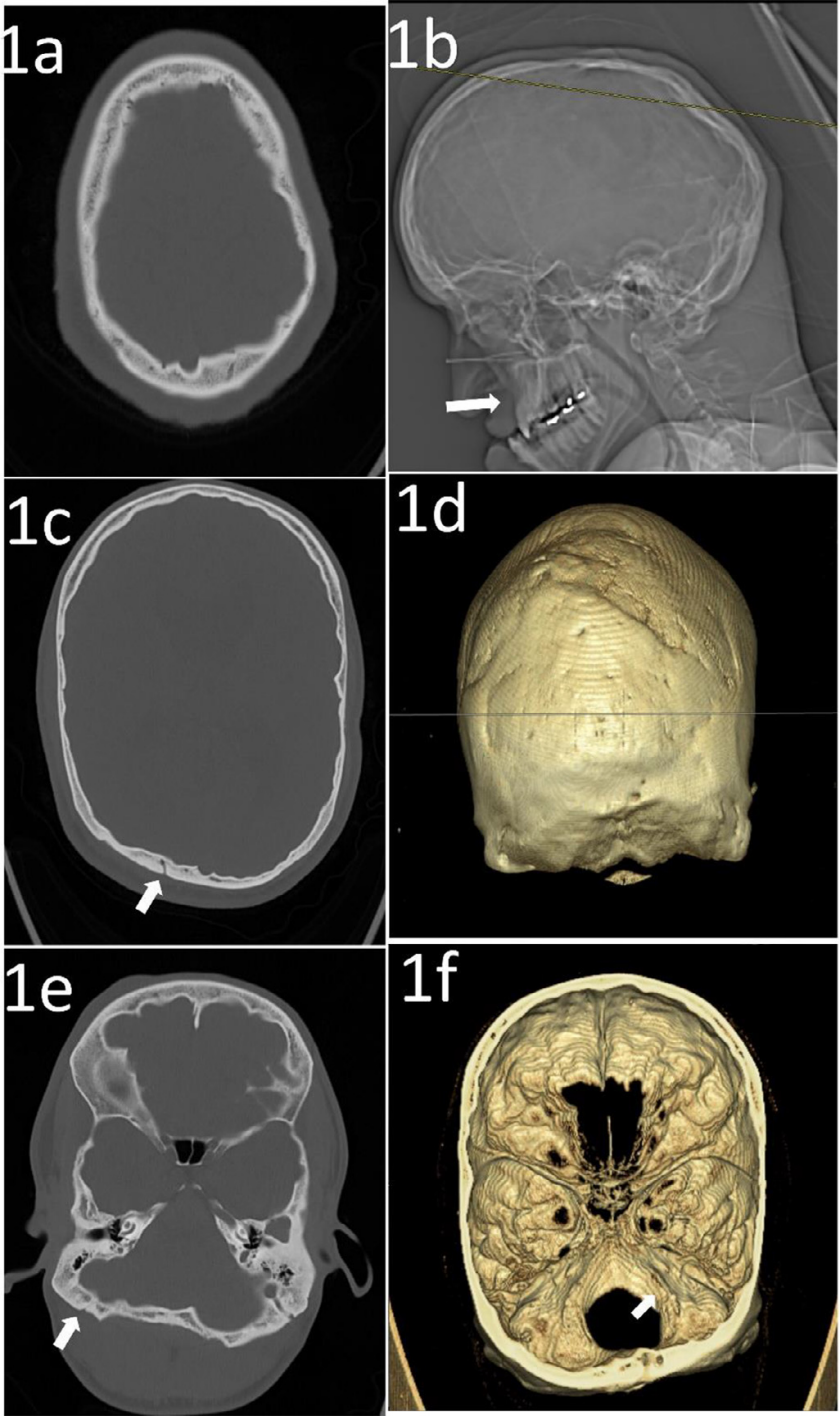
A 21-year- old male with a known Pfeiffer Syndrome presented following a trauma without fractures. Axial brain CT without IV contrast in bone algorithm at multiple levels (1A) through (1E) demonstrates absence of coronal and sagittal sutures. The Lambdoid suture is faintly visualized, white arrows (1C) and (1E). A scout view (1B) demonstrates prognathism due to maxillary hypoplasia. (1D) and (1F) a 3D reconstruction of the skull. (1D) Again, showing the absence of the coronal, sagittal, and lambdoid sutures. (1F) shows an anomalous Jugular foramen.

**Fig. 2:**
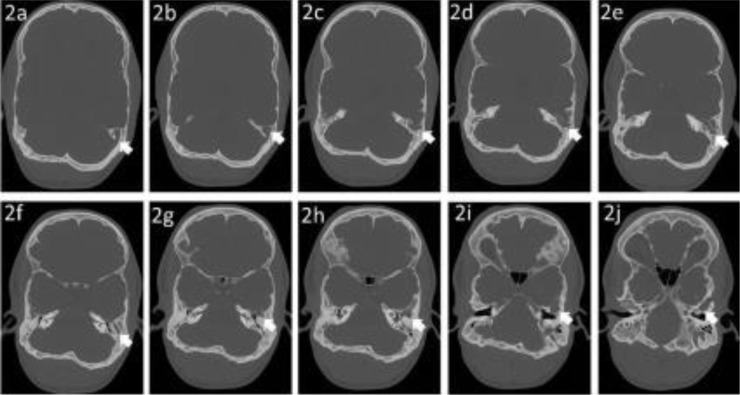
A 21-year- old male with a known Pfeiffer Syndrome presented following trauma. Axial brain CT without IV contrast in bone algorithm at multiple levels showing sections in the craniocaudal direction (2A) through (2J) following the left sided anomalous venous channel.

**Fig. 3:**
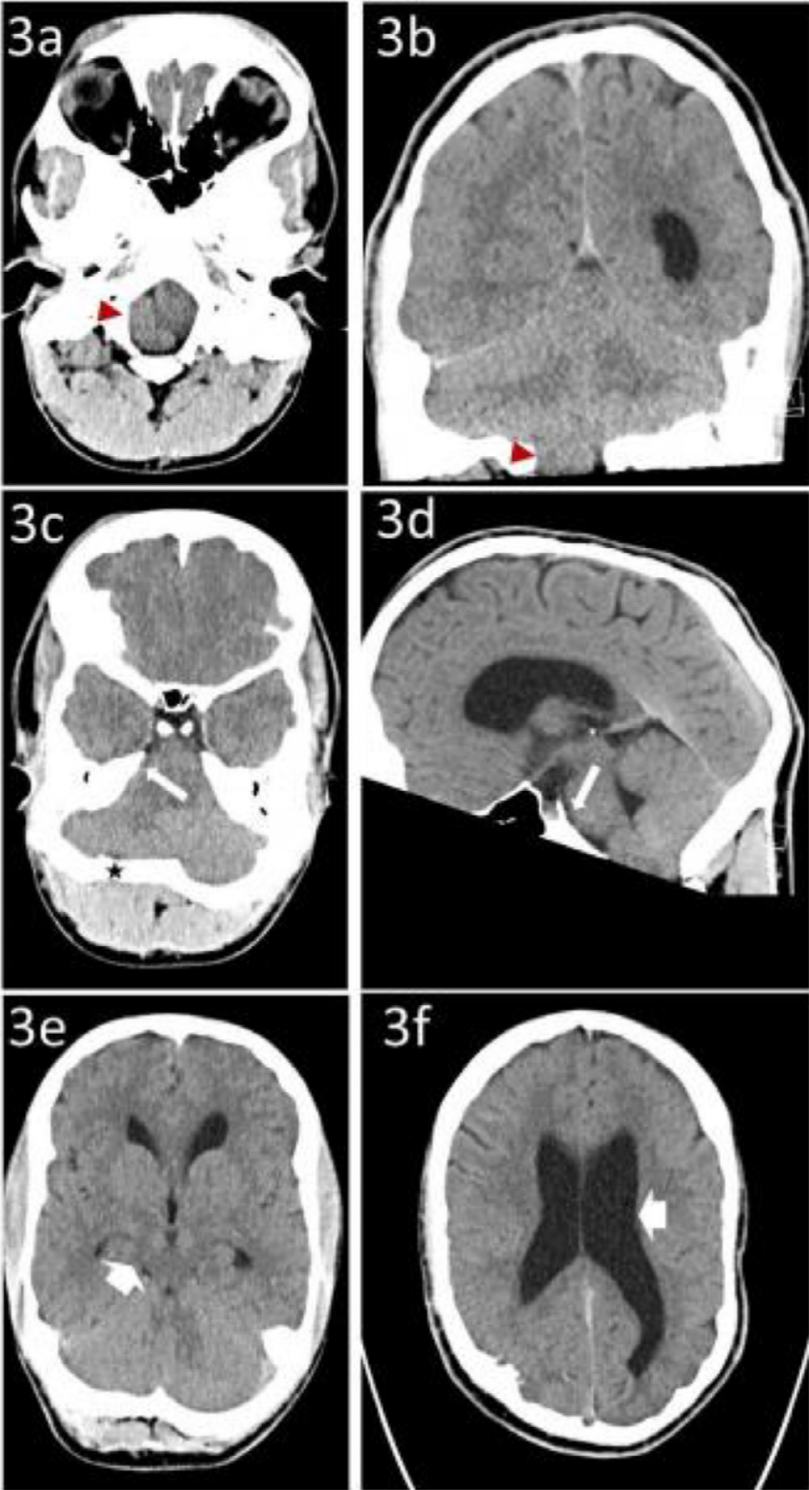
A 21-year-old male with a known Pfeiffer Syndrome presented following trauma. Brain CT without IV contrast (3A), (3C), and (3E) in the axial plane. (3B) in the coronal plane and (3D) in the sagittal plane. (3A) and (3B) demonstrate crowding of the foramen magnum with extension of the right cerebellar tonsil through the foramen magnum, red arrow head. (3C), and (3D) shows prepontine cistern effacement, white long arrow. (3C) shows narrowing of the occipital fossa, black star. (3E) quadrigeminal cistern effacement is demonstrated marked by the short white arrow. (3F) shows bilateral enlargement of the lateral ventricles, short white arrow.

There is midface hypoplasia showing a hypoplastic maxilla with prominent underbite causing prognathism or forward protrusion of the lower jaw resulting in overlapping of teeth, as visualized on scout imaging. There is nonvisualization of the expected coronal and sagittal sutures and only faint visualization of the lambdoid sutures, suggestive of craniosynostosis (Fig. 1). The expected jugular foramina are not visualized. Additionally, there is an anomalous venous channel extending from the region of the left sigmoid sinus into the mastoid portion of the left temporal bone and out of the base of the skull adjacent to the left TMJ (Fig. 2). There is a diminutive posterior fossa and extension of the cerebellar tonsils below the level of foramen magnum into the cervical canal with effacement of the prepontine cistern and cisterna magna. In addition, there is enlargement of the ventricular system suggesting mild degree of obstructive hydrocephalus likely to do obstruction at the level of Foramen of Luschka (Fig. 3). The constellation of visualized skull abnormalities and resulting physiologic consequences is consistent with Pfeiffer syndrome. Given the patient's stated age and normal intelligence he likely has a nonsevere subtype (type 1, classic) [2].

## Discussion

Diagnosis is usually made in the newborn period or later based on the presence of craniosynostosis and short, broad thumbs and big toes. Premature fusion of coronal and lambdoid sutures and to lesser degree sagittal sutures cause characteristic skull shape. Pfeiffer syndrome is rarely detected in the prenatal period. As few as 6 cases have been identified prenatally, usually by ultrasound showing presence of cloverleaf skull deformity [11,12]. Other sonographic signs include brachycephaly, acrocephaly, craniosynostosis, hypertelorism, and hand feet anomalies, but are not as reliably seen.

Pfeiffer syndrome displays large clinical variability even within the same pedigree. This results in increased reliance on clinical genetic data, specifically looking for FGFR1 exon 7, FGFR 2 exon 8 and 10, or FGFR3 exon 7 mutation. Genetic counseling should be considered for affected patients prior to conceiving as Pfeiffer syndrome's autosomal dominant inheritance pattern results in 50% chance of transmission. Advanced paternal age is a noted risk factor for de novo or sporadic mutation [13].

As a specific syndrome all Pfeiffer subtypes display craniosynostosis, midface deficiency, hypertelorism, exorbitism, downslanting palpebral fissures, choanal stenosis or atresia, broad and short great toes, broad thumbs, and variable brachydactyly (bone shortening of digits) [6]. Patients can also display upper airway obstruction due to midface hypoplasia and nasal obstruction, mental retardation, aqueductal stenosis causing hydrocephalus, cerebellar and brain stem herniation, and infrequently abdominal abnormalities including hydronephrosis, pelvic kidneys and hypoplastic gallbladder [4].

Pfeiffer syndrome can be subdivided into three subtypes with overlap: Classic or type 1 is the most mild with brachycephaly (flat head syndrome), midface hypoplasia, finger and toe abnormalities, normal lifespan and intellect, with good outcome post surgery. Type 2 is specifically characterized by trilobolated or cloverleaf skull, extreme proptosis, finger and/or toe abnormalities, elbow ankylosis and/or synostosis, beak-shaped nose, and developmental and neurologic complications (ie, hydrocephalus) due to limited brain growth. Type 3 is classified by the more severe abnormalities of type 2 but without the characteristic cloverleaf skull (Table 1). Types 2 and 3 are characterized by shortened life expectancy due to neurologic and respiratory problems [4,14].

**Table 1:** Pfeiffer subtypes.

*Pfeiffersubtypes*	*Characteristic features*
Type 1 (classic)	More mild - Bicoronal craniosynostosis
Type 2	More severe - particularly CNS involvement (hydrocephalus), Additional suture involvement, Characteristic Cloverleaf skull shape
Type 3	More severe - particularly CNS involvement (hydrocephalus), Lack of cloverleaf skull

The five types of acrocephalosyndactyly syndromes make up Pfeiffer syndrome's differential among a few other options. These include the most common craniosynostosis syndrome - Apert syndrome or type I acrocephalosyndactyly, Crouzon syndrome or type II acrocephalosyndactyly, Saethre-Chotzen syndrome or type III acrocephalosyndactyly, Muenke syndrome, and Jackson-Weiss syndrome [14]. Pfeiffer syndrome is genetically and nosologically distinct from Apert syndrome. Although they share some phenotype similarities such as tracheal cartilaginous sleeve (TCS), an airway malformation lacking distinct tracheal rings and rather one continuous cartilaginous segment from subglottis to carina is present. This is also seen in Crouzon syndrome, which is phenotypically very similar to Pfeiffer syndrome, but lacking the limb anomalies. Pfeiffer syndrome shares some phenotype similarities to Muenke syndrome, however this syndrome is typically due to a FGFR3 rather than FGFR1 or 2 mutation. Saethre-Chotzen and Jackson-Weiss syndromes share similar broad toes at birth and have been confused for Pfeiffer syndrome [1,14].

As mentioned, type 1 Pfeiffer syndrome has a good prognosis with normal life expectancy and intellect, although long term follow up has been poor due to paucity of cases. This prognosis is assuming that the patient undergoes multiple staged surgeries starting within the first year of life. Type 2 and 3 Pfeiffer syndrome have poor prognosis with neurodevelopmental complications and childhood death being common. Severity of syndrome is the most relevant factor in prognosis. Craniofacial appearance improves with age. A study reviewing clinical course of seven children with type III Pfeiffer syndrome noted that even given severe manifestations, aggressive medical and surgical management can result in favorable outcomes [1,^14^,15].

Primary treatment is surgery of craniofacial abnormalities in a set of staged corrective procedures with the aim of brain decompression to ensure adequate intracranial volume and to widen the nasopharynx by advancement of naso-maxillary-zygomatic complex. These are done with fronto-orbital and Le Fort III advancement starting in the first year of life (as early as three months of age) with suturectomy to release the synostotic sutures and open up intracranial space for bone growth. The second stage involves mid-facial surgery to reduce exophthalmos and midfacial hypoplasia. If TCS is present, patients usually eventually require tracheostomy to treat the side effect of obstructive sleep apnea due to combined maxillary hypoplasia, choanal stenosis, and macroglossia. Cosmetic surgical repair can be done for other abnormalities relevant to each patient's phenotype. An interprofessional care team involving neurosurgery, pediatrics, plastics, and skilled nursing among others is the best approach for favorable patient outcome with a plan tailored to the individual patient's strengths and weaknesses [15,16].

**Table 2:** Summary table.

ETIOLOGY	Autosomal Dominant classically, but also de novo sporadic mutations of FGFR-1 and 2 are documented
INCIDENCE	1 in 100,000, second most common acrocephalosyndactyly
GENDER RATIO	1:1
AGE	
PREDILECTION	Congenital, can be diagnosed as early as 20 wk gestation due to sonographic features including broad thumb and craniosynostosis
RISK FACTORS	Advanced paternal age, family history
TREATMENT	Multiple staged surgeries including suturectomy and brain decompression, cosmetic surgical repair, ventriculoperitoneal shunting, tracheostomy
PROGNOSIS	-Type 1 has a relatively good prognosis with normal intelligence and lifespan.
-Type 2 and 3 are more variable, however death in childhood is common due to neurodevelopmental complications	
IMAGING FINDINGS	Variable degree of craniosynostosis (cloverleaf skull, brachycephaly), Calvarial thinning, Small posterior fossa, Cerebellar tonsil herniation, Bony spiculations,, Dental abnormalities, Maxillary hypoplasia

**Table 3:** Differential diagnosis.

Disease	Clinical findings	Imaging findings (mainly bony)
Pfeiffer Syndrome	Ankylosis of elbows, Broad thumbs and toes, Hypertelorism, Proptosis, Midface hypoplasia	Bicoronal craniosynostosis with multiple other sutures often involved (cloverleaf skull), Ventriculomegaly
Apert Syndrome	Syndactyly (mitten hand), Hypertelorism, Flat and Bulbous nose, Underbite, Strabismus, Proptosis, Midface hypoplasia	Bicoronal synostosis, Maxillary Hypoplasia,, Ventriculomegaly, malformation of corpus callosum, limbic structures, gyral abnormalities, hypoplastic WM, heterotopic GM
Crouzon Syndrome	Beaked nose, Proptosis, Hypertelorism, Midface hypoplasia	Bicoronal synostosis, Maxillary hypoplasia, Cervical spine abnormalities
Saethre Chotzen Syndrome	Towering/turricephalic forehead, Low set hairline, Ptosis, Partial syndactyly, Small ear pinna with prominent crus, Strabismus	Craniosynostosis of coronal, lambdoid, and/or metopic sutures
Muenke Syndrome	Hypertelorism, Macrocephaly, High narrow palate	Fusion of carpal and tarsal bones, Thimble like phalanges, Cone shaped epiphysis
Jackson Weiss Syndrome	Hypertelorism, proptosis, syndactyly (almost exclusively feet), midface hypoplasia, ptosis	Craniosynostosis, Ventriculomegaly, flat occiput, malformation of metatarsals, tarsals, and calcanei

## Conclusion

Pfeiffer syndrome is a rare disease with both specific bony abnormalities and variant phenotypes from patient to patient that make it a distinct acrocephalosyndactyly syndrome. Given the paucity of follow up, this case provides a unique assessment of long-term viability of pediatric surgical modifications and the body's physiological adaptation to accommodate them. Imaging features consist of characteristic craniosynostosis patterns including brachycephaly, acrocephaly, or cloverleaf shaped skull, brain stem herniation, obstructive hydrocephalus, pelvic kidneys, compressed gallbladder, and hydronephrosis.

## Patient consent

Did the author obtain written informed consent from the patient for submission of this manuscript for publication? (no.)

## Human and animal rights

No experiments conducted.
